# ARL4C Regulates the Progression of Clear Cell Renal Cell Carcinoma by Affecting the Wnt/*β*-Catenin Signaling Pathway

**DOI:** 10.1155/2022/2724515

**Published:** 2022-06-21

**Authors:** Peizhi Zhang, Yingkun Xu, Shaoan Chen, Zicheng Wang, Leizuo Zhao, Chen Chen, Weiting Kang, Rongyu Han, Jiechuan Qiu, Qingliang Wang, Han Gao, Guangzhen Wu, Qinghua Xia

**Affiliations:** ^1^Department of Urology, Shandong Provincial Hospital, Cheeloo College of Medicine, Shandong University, Jinan 250021, China; ^2^Department of Endocrine and Breast Surgery, The First Affiliated Hospital of Chongqing Medical University, Chongqing 400042, China; ^3^Department of Urology, Shandong Provincial Hospital Affiliated to Shandong First Medical University, Jinan 250021, China; ^4^Department of Urology, Dongying People's Hospital, Dongying 257000, China; ^5^Department of Urology, Liaocheng People's Hospital Affiliated to Shandong University, Liaocheng 252000, China; ^6^Department of Urology, The First Affiliated Hospital of Dalian Medical University, Dalian 116011, Liaoning, China

## Abstract

**Purpose:**

To investigate the expression of the ADP-ribosylation factor (ARF)-like proteins (ARLs) and ARL4C in clear cell renal cell carcinoma (ccRCC) based on bioinformatics analysis and experimentally determine the effect and mechanism of ARL4C on cellular properties involved in ccRCC progression.

**Methods:**

After downloading the data of cancer patients from the TCGA database, we used various bioinformatics analysis websites and methods to analyze the expression and function of ARLs and ARL4C. The differential expression of ARL4C in clinical renal cancer tissues versus adjacent normal tissues was further verified using immunohistochemistry and real-time quantitative reverse-transcription (qRT-PCR). qRT-PCR was used to explore the expression of ARL4C mRNA in normal renal cells versus different ccRCC cell lines, and the protein expression of ARL4C was further verified using western blotting. CCK-8, colony formation, and EdU assays were used to determine the effect of ARL4C knockdown on ccRCC cell proliferation. We also used wound healing and Transwell assays to analyze the changes in ccRCC cell migration and invasion following ARL4C knockdown. Finally, we used western blotting to probe the molecular mode of action of ARL4C in ccRCC cells after exposure to Wnt signaling pathway agonists.

**Results:**

Biological function analysis showed that methylation of ARL4C and changes in immune cell infiltration and targeted drug sensitivity caused by altered ARL4C expression affected the prognosis of ccRCC. Further bioinformatics analysis suggested that the expression of ARL4C mRNA was increased in ccRCC, and this was associated with a poor prognosis in ccRCC patients. Increased expression of ARL4C was further verified using qRT-PCR and western blotting of human ccRCC tissue samples. Downregulation of ARL4C significantly inhibited the proliferation, migration, and invasion of ccRCC cells, and activation of the Wnt/*β*-catenin pathway promoted the expression of ARL4C. As an essential downstream effector of the Wnt signaling pathway, ARL4C increased the expression of cyclin D1 and c-myc, thereby increasing the ability of the cells to undergo epithelial-mesenchymal transition (EMT) and ccRCC progression.

**Conclusions:**

As a critical factor in the Wnt/*β*-catenin pathway, ARL4C regulates EMT and progression in ccRCC.

## 1. Introduction

Cancer is the first or second most common cause of death in people over the age of 70 in more than 60% of the countries globally, according to the latest World Health Organization (WHO) global health statistics [1]. Due to regional differences in many risk factors such as living environment, lifestyle, and ethnicity, the incidences and mortality rates of renal cell carcinoma (RCC) vary widely worldwide. Although the diagnosis and treatment of RCC have gradually improved in recent times, RCC remains one of the main cancers threatening human life and health [2]. Clear cell RCC (ccRCC) accounts for 75% of all primary RCCs and is the most common subtype of the numerous pathological subtypes [3]. As a result of the rapid development of medicine in recent decades, there are many treatments for ccRCC in clinical practice. The most widely used and effective treatment is still surgery. However, for patients with a high tumor stage or poor physical condition, the possibility of tumor recurrence and metastasis is still high even when they receive surgical treatment. For advanced renal cancer patients with distant tumor metastasis, only half of the patients survive for more than one year, and only 10% of patients survive for more than five years [4, 5]. Although the survival rate for patients with metastatic RCC has increased significantly due to continuous research and development of antiangiogenic drug-targeted therapy and immunotherapy, long-term drug resistance generally leads to treatment failure [6–8]. Therefore, identifying and exploring new molecular markers that cause cancer and exploiting these as targets for gene therapy has become a high-priority topic in current cancer treatment research.

ARL4C is one of more than 150 members of the GTP-binding proteins (*G* proteins) superfamily, which includes five subfamilies, namely, Rho, Ras, Rab, Ran, and Arf [9]. These subfamilies are further subclassified according to their molecular composition and biological activities, and the Arf family contains three different protein subgroups: ARFs, SARs, and ARLs. ARLs [ADP-ribosylation factor (ARF)-like proteins] are currently the most extensively studied subgroup. More than 20 forms of ARLs have been discovered, and there is a considerable degree of identity in terms of the primary protein sequence and structure among the subgroup members, which also leads to similar biological functions among the various members [10]. Although related studies have provided preliminary confirmation that members of this family act as regulators of actin remodeling and membrane transport, thereby affecting the secretion, endocytosis, and phagocytosis of relevant molecules within and between cells, the biological effects of each molecule's function are unclear [11]. At present, the research on ARL4C is at a relatively early stage. In terms of molecular structure, ARL4C [ADP-ribosylation factor (ARF)-like protein 4c] has a primary protein sequence similar to other members of this subgroup. In addition, the proteins in this subgroup share the same nuclear localization signal, and all of them have unusually high guanine nucleotide exchange rates [12]. Further research on the molecular structure of ARF family proteins has found that they contain an amphipathic helix and a specific lipid modification site at the *N*-terminus. They also have multi-clusters containing amino acid residues at the *C*-terminus. In the middle region of the molecule, there are two switch regions called switch1 and switch2, and inter-switch regions of different lengths between the switch regions [13]. The switch and inter-switch regions undergo conformational changes upon GTP binding, leading to changes in molecular conformation and altered biological functions [14]. However, the molecular structures differ among ARF family proteins, and these differences also determine the differential functions of each member of the ARF protein family in cells. The inter-switch region in ARL4C protein is longer than that in other ARF family proteins, and this prevents the ARL4C protein from forming a retractable conformation in the GDP-bound state [15]. Unlike other ARF family proteins, ARL4C only produces changes in molecular structure when the switch region is shifted, and this structural change depends on the interaction of the ARL4C protein with GDP and GTP. In terms of biological functions, the members of this subfamily are mostly located on cell membranes, including cell surface membranes of secretory and endocytic vesicles and intracellular organelle membranes, which is why members of this family are involved in regulating membrane trafficking. Specifically, the ARL4C protein in cells is present on the cell surface membrane as well as being distributed in the cytoplasm and nucleus. This change in intracellular distribution often depends on the presence or absence of the C-terminus in the molecular structure of ARL4C [12, 16]. This may reflect that the C-terminus of ARL4C can to a certain extent determine its localization to the nucleus. It is well known that the progression of cancers involves remodeling of cell membranes and molecular structural changes in key regulatory factors. The expression of ARL4C varies in different tissues as well as in different pathological types of tumors [17–19]. As a tumor-related gene, ARL4C is associated with lung cancer, colorectal cancer, gastric cancer, testicular cancer, melanoma, primary human glioblastoma, and ovarian cancer, among others. Although recent research has shown increased expression of ARL4C in kidney renal clear cell carcinoma (KIRC) tissues, the specific mechanism of action of ARL4C in the progression of ccRCC has yet to be elucidated [20].

In the present study, ARL4C expression was elevated in renal cancer, and proliferation, migration, and invasion of ccRCC cells could be inhibited by downregulation of ARL4C. Activation of the Wnt/*β*-catenin pathway was found to increase the expression of ARL4C. As an essential downstream effector of the Wnt signaling pathway, ARL4C upregulated the expression of cyclin D1 and *c*-myc, thereby affecting the epithelial-mesenchymal transition (EMT) of ccRCC cells. These findings suggest that ARL4C promotes ccRCC progression by the mechanisms identified above. ARL4C acts as an oncogene in ccRCC, and an in-depth study of its mode of action may result in the identification of new targets and prognostic markers for gene therapy of renal cancer.

## 2. Materials and Methods

### 2.1. ccRCC Tissue Samples

Forty-two pairs of human ccRCC and adjacent normal tissue samples were collected from patients at Shandong Provincial Hospital between 2019 and 2021. For all samples and information collected, the patients provided written consent. We analyzed these 42 sample pairs using real-time quantitative reverse-transcription polymerase chain reaction (qRT-PCR). In addition, we collected three pairs of samples of ccRCC and adjacent normal tissue from KIRC patients. The tissue specimens were fixed, embedded in paraffin, and then cut into tissue sections. This study was approved by the Ethics Committee of Shandong Provincial Hospital. The research adhered to the principles of the Declaration of Helsinki and those of the World Medical Association.

### 2.2. Cell Lines, Antibodies, And Reagents

The cell lines used in this study included ACHN, A498, 786-O, and HK2, purchased from the Chinese Academy of Sciences Cell Bank (Shanghai, China). These cells were cultured according to established procedures in a medium containing 10% fetal bovine serum with penicillin/streptomycin in a 37 incubator with a humidified 5% carbon dioxide atmosphere.

Rabbit anti-*β*-actin (ab8227; Abcam) was used as a reference protein antibody. The target protein antibodies and Wnt pathway-related antibodies included rabbit anti-ARL4C (ab122025, Abcam), anti-c-myc (ab32072, Abcam), and anti-cyclin D1 (ab40754, Abcam). The EMT-related protein antibodies used in this study were anti-E-cadherin, anti-N-cadherin, and anti-vimentin (Proteintech, Wuhan, China). The Wnt agonist 1 powder (Selleck Chemicals, Shanghai, China) was dissolved according to the manufacturer's instructions and added to the medium containing ccRCC cells for 24 h at 37°C.

### 2.3. Bioinformatics Analysis

The mRNA expression data and clinical datasets of KIRC patients (539 tumor tissues and 72 normal tissues) used in this study were obtained from The Cancer Genome Atlas (TCGA) database (https://portal.gdc.cancer.gov/). We then used the TCGA database to probe for differential expression of 22 ARL genes in renal cancer and normal kidney tissues and utilized the “pheatmap” expansion package to generate the heatmap [21]. Based on multiple extension packages of the *R* language, we performed statistical analyses on these 22 ARL genes. After univariate Cox analysis of the ARL genes, we generated a forest plot. Lasso regression analysis was performed using “glmnet” expansion packages. The survival curve was generated using the “survival” expansion packages to screen ccRCC prognosis-related genes. We then combined the Cox coefficient and gene expression to evaluate the risk score and drew the corresponding survival curve according to the risk score level. Receiver-operator characteristic (ROC) curves were analyzed and plotted with the survival ROC software package. GSCALite was used to study the effect of the methylation level of ARL genes on the survival prognosis of tumor patients, explore the relationship between ARL genes and drug sensitivity, and generate a heatmap for visual display. The powerful function of the ImmuCellAI website allows evaluation of the relationship between different immune cell types and ARL genes in KIRC. Next, we analyzed the infiltration of 24 types of immune cells in pan-cancer, and the analysis results were displayed as a heat map using the “pheatmap” expansion package. Finally, we used UALCAN, an online tool, to investigate the expression of ARL4C in pan-cancer and its correlation with the clinicopathological features of ccRCC patients.

### 2.4. Immunohistochemistry

The ccRCC and adjacent normal tissue samples were sectioned after embedding in paraffin and were then deparaffinized with xylene and hydrated with graded alcohol. We incubated the pathological sections with an anti-ARL4C antibody overnight at 4°C. After one day, the pathological sections were incubated with a biotin-conjugated secondary antibody for 30 minutes. Target protein expression was detected using a DAB kit (ab64238; Abcam), and the nuclei were stained with hematoxylin (ab143166; Abcam). We recruited two pathologists to evaluate and score the pathological sections. Finally, the corresponding H-scores were objectively calculated and counted.

### 2.5. qRT-PCR Assay

An RNA extraction kit provides a simple and effective method for extracting RNA from tissues or cells. We used this kit to extract total RNA from ccRCC tissues and cells while strictly following the manufacturer's instructions. Next, we reverse-transcribed the pre-extracted RNA into cDNA using EvoM-MLVRT master mix. We then mixed the reagents for qRT-PCR detection according to the manufacturer's instructions of the SYBR^®^ Green Premix Pro Taq HS qPCR Kit and amplified the target sequences using a LightCycler 480II device (Roche, Switzerland). The above reagents were purchased from Accurate Biotechnology (Hunan, China). *β*-Actin was used as an endogenous reference to normalize RNA expression. After obtaining the crossing-point value, we used the 2^−ΔΔCt^ method to analyze the relative mRNA expression. The primer sequences for *β*-actin were as follows: *β*-actin-F, 5′-TGGCACCCAGCACAATGAA-3′ and *β*-actin-R, 5′-CTAAGTCATAGTCCGCCTAGAAGCA-3′. The primer sequences for ARL4C were as follows: ARL4C–F, 5′-GCAGTAAAGTAAAGCCCTGTGGTG-3′ and ARL4C-R, 5′-GGTCAGAGACGAAACGGGCTA-3′.

### 2.6. Western Blot Assay

Total protein from ccRCC cells was extracted using RIPA lysis buffer (Solarbio). Next, a bicinchoninic acid (BCA) reagent test kit (Solarbio) was used to determine the protein concentration, and the proteins of each sample were separated using sodium dodecyl sulfate-polyacrylamide gel electrophoresis (Epizyme) and transferred to polyvinylidene fluoride (PVDF) membranes. After blocking with 5% nonfat dry milk, the membranes were incubated with primary antibodies overnight, followed by incubation with peroxidase-conjugated secondary antibodies. Finally, we used a chemiluminescence detection system (Amersham^™^ Imager 600; General Electric, Fairfield, CT, USA) to visualize the immunoreactive bands corresponding to the target proteins and endogenous reference proteins.

### 2.7. Establishment of ARL4C-Knockdown ccRCC Cells

We commissioned GenePharma (Shanghai, China) to design and synthesize small interfering (si)-RNA directed against ARL4C (si-ARL4C) and negative control vector (si-NC), which were then transfected into human ccRCC cells using Lipofectamine 3000 (Thermo Fisher, MA, USA). After 48 h of treatment, we verified the transfection efficiency using real-time PCR and western blotting.

### 2.8. Cell Counting Kit-8 Assay

We seeded ccRCC cells of the si-ARL4C and si-NC groups in triplicate into 96-well plates at a density of 4,000 cells/well. The cells were cultured for 24, 48, 72, and 96 h. After removing the medium, each well was washed with phosphate-buffered saline (PBS), and the Cell Counting Kit-8 (Dojindo) reagent was mixed in a serum-free medium. The reagent concentration was diluted to 10%, and then 100 *μ*L of this mixture was added to each well. After incubating the 96-well plate for 60 minutes at 37°C, a microplate reader was used to measure the absorbance at 450 nm.

### 2.9. Colony Formation Assay

After counting the ccRCC cells of the si-ARL4C and si-NC groups under a light microscope, the cells were cultured in six-well plates at a density of 500 cells/well. The plates were shaken to disperse the cells evenly on the bottom of the plate, and the cells were then cultured in an incubator at 37°C. When individual colonies became visible, the original medium was removed, and the plates were washed twice with PBS. After being fixed in 4% paraformaldehyde for 30 minutes, the cells were stained with 0.1% crystal violet for 10 minutes at room temperature. Finally, we used Image-Pro Plus software (Media Cybernetics, Bethesda, MD, USA) to count the number of colonies.

### 2.10. Ethynyl-2'-Deoxyuridine (EdU) Assay

The ccRCC cells of the experimental and control groups in the logarithmic growth phase were seeded into a 24-well plate. EdU was added to the medium at a final concentration of 50 *μ*M. The cells were placed in a 37°C incubator for 3 h and then fixed with 4% paraformaldehyde. After permeabilization of cells with 0.5% TritonX-100 for 15 minutes, the cells were stained with Hoechst for 30 minutes. All the above procedures were carried out at room temperature. The cells were then imaged using a microscope (Olympus, Tokyo, Japan), and the images were analyzed using Image-Pro Plus software.

### 2.11. Wound Healing Assay

CcRCC cells were seeded into six-well plates after treatment with siRNAs. The cell layer was scraped with a sterile 200 *μ*L pipette tip when the confluency of the ccRCC cells reached 95%, after which the detached cells were removed by washing with PBS, and serum-free medium was added to the plates. The cell monolayers were then imaged at 0 and 24 h after scratching and quantified using Image-Pro Plus to quantify the wound healing rate.

### 2.12. Transwell Migration and Invasion Assays

Solubilized Matrigel^®^ (Corning, USA) was mixed with serum-free medium according to the manufacturer's instruction and placed into a Transwell chamber (Corning, USA) at 37°C to form a gel. Medium containing 10% fetal bovine serum (FBS) was added to the bottom chamber, while no FBS was present in the medium in the upper chamber. The difference in the FBS concentration between the upper and lower chambers induced ccRCC cell movement from the upper chambers to the bottom chambers. After culturing the cells for 24 hours, the chamber inserts were removed, a cotton swab was used to wipe the bottom of the Transwell chamber, and the cells were washed with PBS and fixed with paraformaldehyde. Cells on the bottom and rear of the chamber were stained with crystal violet and counted. The difference in the experimental approach between the Transwell migration and invasion assays is that no Matrigel^®^ was added to the bottom of the Transwell chamber in the migration assay.

### 2.13. Statistical Analysis

All results are based on three independent experiments and presented as mean ± standard deviation (SD). GraphPad Prism 8 software was used for the statistical analysis of the data. Pearson's *χ*-test was used to correlate the immunohistochemical staining results with the pathological parameters. Student's *t*-test was used to determine between-group differences. One-way ANOVA was used to analyze differences between more than two groups of patients. *P* < 0.05 was considered to indicate statistical significance.

## 3. Results

### 3.1. Expression of ARL Genes in KIRC and Univariate Cox Analysis

**Figure 1:**
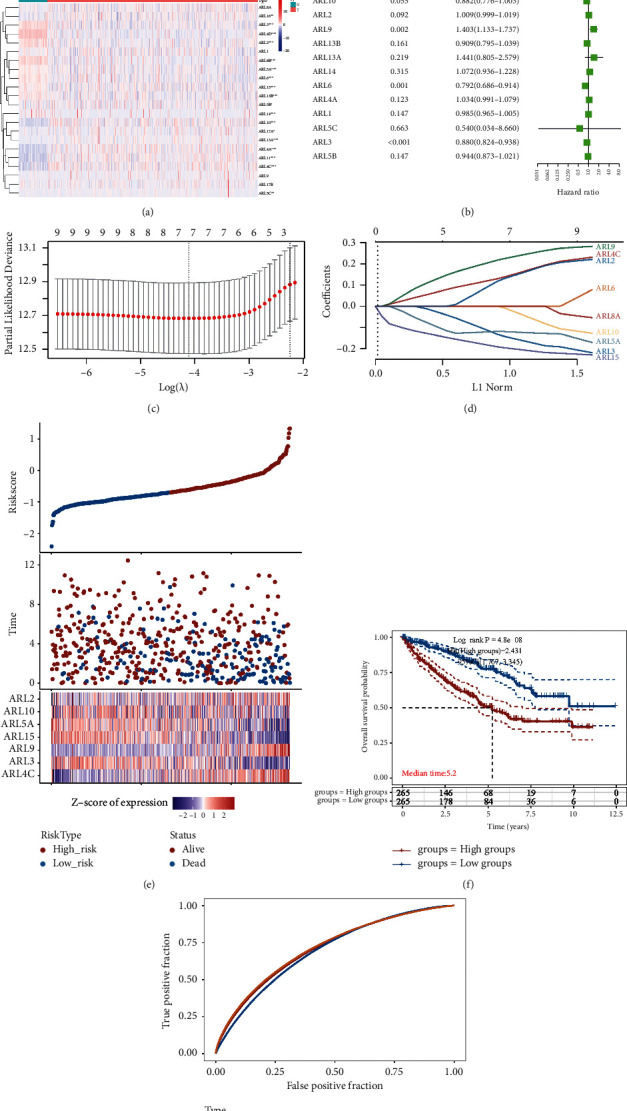
Expression of ARL family genes and establishment of risk models in KIRC. (a) The differential expression of 22 ARL genes in KIRC versus normal kidney tissues. Red and blue colors in the heat map indicate high and low gene expression, respectively. The darker the color of red, the higher the gene expression; the darker the color of blue, the lower the gene expression. (b) Univariate Cox regression analysis of 22 ARL genes. (c-d) LASSO regression curve analysis and cross-validation of ARL genes. (e-f) Risk scores were calculated from linear combinations of Cox coefficients and gene expression. We divided the KIRC patients into high-risk and low-risk groups based on the best cutoff value, and the Kaplan–Meier survival curve was generated, (*P*=4.8*e* − 08). (g) Using the ROC curve to predict 3-year, 5-year, and 7-year survival rates. ^*∗*^*P* < 0.05, ^*∗∗*^*P* < 0.01, ^*∗∗∗*^*P* < 0.001, and ^*∗∗∗∗*^*P* < 0.0001.

The TCGA database contains data on 539 KIRC tumor tissues and 72 normal kidney tissues. We downloaded the mRNA expression data and clinical characteristics of these samples. We applied *R* language to generate a heatmap illustrating the expression of 22 ARL genes in KIRC and normal kidney tissues (Figure 1(a)). Statistical analysis showed that the expression of 18 ARL4C genes was substantially different between normal kidney and KIRC tissues, suggesting that ARL proteins may be involved in the occurrence of KIRC. We then used 22 ARL genes to perform a univariate Cox retrospective analysis in KIRC patients, and we generated a forest plot (Figure 1(b)). Under the premise of *P* < 0.05, the hazard ratio (HR) value was used to assess the importance of ARL genes in KIRC. The HR values of ARL4C and ARL9 were greater than 1, indicating that these genes act as risk factors in the progression of KIRC. Conversely, four genes, namely, ARL5A, ARL6, ARL15, and ARL3, had HR values of less than 1, indicating that they act as protective factors and inhibit the occurrence and development of KIRC. Finally, we further validated these findings using LASSO regression analysis and cross-validation, and we calculated a risk score based on Cox coefficients and gene expression (Figures 1(c) and 1(d)). Taking the median of the risk score as the cutoff value, we divided the KIRC patients into high-risk and low-risk groups (Figures 1(e) and 1(f)), and the survival curve was generated and verified using the ROC curve (Figure 1(g)). We found that the group with high-risk scores had a worse prognosis.

### 3.2. Immune Infiltration, Methylation, And Drug Sensitivity of ARL4C Genes in KIRC and Pan-Cancer

**Figure 2:**
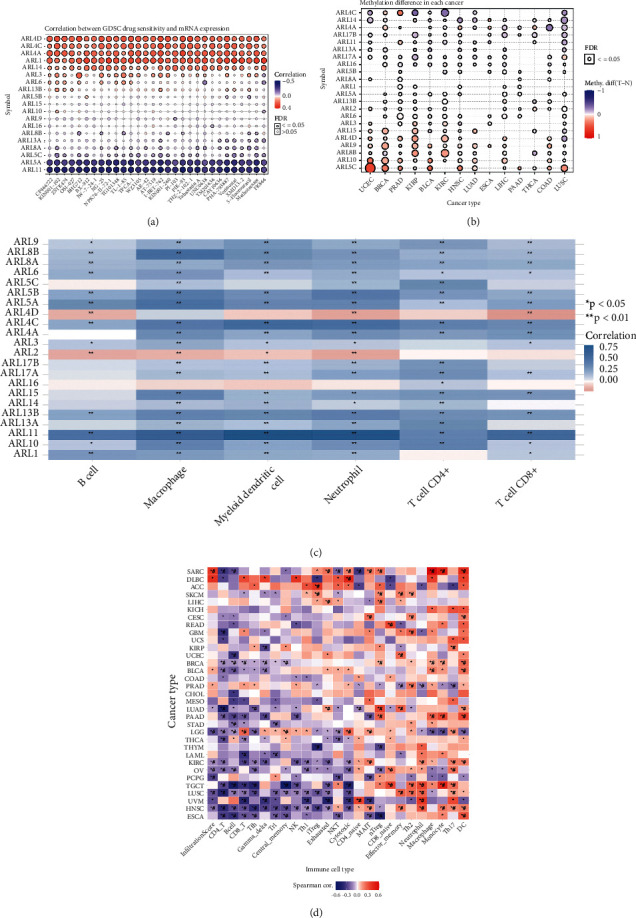
Immune infiltration, methylation, and drug sensitivity of ARL4C genes in KIRC and pan-cancers. (a) Correlation analysis between ARL mRNA expression and GDSC drug sensitivity. Red and blue colors indicate that gene expression was positively or negatively correlated, respectively, with drug sensitivity. The darker the color, the higher the correlation. (b) Methylation of ARL genes in different tumor types. Red indicates hypermethylation of the gene in the tumor, and the darker the color, the higher the methylation; blue indicates hypomethylation in the tumor, and the darker the color, the more pronounced the inhibition of methylation. (c) Correlation between different immune cells and ARL gene expression in KIRC patients. Blue represents an increase in immune infiltration with increasing gene expression, whereas red indicates that the correlation was negative. The darker the color, the higher the correlation. (d) Heat map showing the correlation of ARL gene expression with the infiltration of various immune cells in different types of pathological tumors. ^*∗*^*P* < 0.05; #: false discovery rate (FDR) ≤ 0.05.

We downloaded the drug data from the GDSC database, and we explored the correlation between ARL mRNA expression and GDSC drug sensitivity (Figure 2(a)). When the sensitivity of drugs increased with gene expression, the image appeared red on the heatmap. In other words, the darker the red color, the higher the gene expression, indicating a greater drug treatment effect. Conversely, the darker the blue color in the heatmap, the more negative the relationship is between gene expression and drug sensitivity, indicating that the drug treatment is less sensitive. The results show that, in KIRC patients, the higher the expression of ARL genes, such as ARL4C, ARL4D, ARL4A, ARL1, and ARL14, and especially the ARL4C and ARL4D genes, the higher the sensitivity of applying targeted drugs, suggesting that these genes may be potential targets of targeted drugs. In contrast, the higher the expression of ARL5A and ARL11 genes, the lower the sensitivity to tumor-targeted drugs. Modification of DNA, RNA, and even proteins by methylation changes the molecular structure. These changes in molecular conformation can lead to inactivation or activation of cancer-related molecules, which affects the prognosis of cancer [22]. In light of this, we compared the methylation status of ARL genes in different tumors versus their corresponding normal tissues (Figure 2(b)). The red color represented a high degree of methylation of this gene in tumor tissue, and the darker the color, the higher the degree of methylation. In contrast, the blue color indicated that methylation in tumor tissue was reduced, and the darker the color, the greater the reduction in methylation. The results show that different genes had disparate methylation levels. Most importantly, we found that the methylation of ARL4C, ARL4D, and ARL8B in KIRC was significantly altered compared with that in normal kidney tissue. Of these, the methylation levels of ARL4D and ARL8B were higher in KIRC tissues than those in normal kidney tissues. Additionally, the methylation level of ARL4C was reduced in KIRC tumors, suggesting that the demethylation of ARL4C may be involved in the progression of KIRC. Several recent studies have indicated that changes in levels of inflammatory mediators caused by the infiltration of immune cells in the tumor microenvironment may cause local inflammatory responses, thereby affecting tumor progression. Immunotherapy targeting tumor immune cells has become a promising option for treating advanced cancers [23]. We explored the correlation between various immune cells and ARL gene expression in KIRC patients (Figure 2(c)). The red color in the heatmap indicated that immune infiltration increased with gene expression, whereas the blue color indicated that immune infiltration decreased with increasing gene expression. The heatmap visually showed that ARL4C expression had a positive association with infiltration of B cells, macrophages, myeloid dendritic cells, neutrophils, CD4^+^ T cells, CD8^+^ T cells, and other immune cells, suggesting that ARL4C may be involved in the infiltration of these immune cell types in the KIRC tumor microenvironment. Finally, we explored the correlation between ARL genes and the infiltration of different immune cells in various pathological types of tumors. We found that the degrees of infiltration of different immune cells in various pathological types of tumors were considerably different. Notably, we found that different types of immune cells play different roles in the progression of KIRC (Figure 2(d)). The infiltration of mucosal-associated invariant *T* cells, neutrophils, dendritic cells, Th17, and other immune cells in KIRC tumors was increased, suggesting that infiltration of these immune cell types may promote KIRC. CD4T, CD8T, Tfh, gamma delta *T*, natural killer, Th1, and cytotoxic *T* cells were reduced in KIRC, suggesting that the infiltration of these immune cells plays a pivotal role in killing or inhibiting the tumors.

### 3.3. Expression of ARL4C in Pan-Cancer and KIRC and Its Relationship with Clinical Features of KIRC Patients

**Figure 3:**
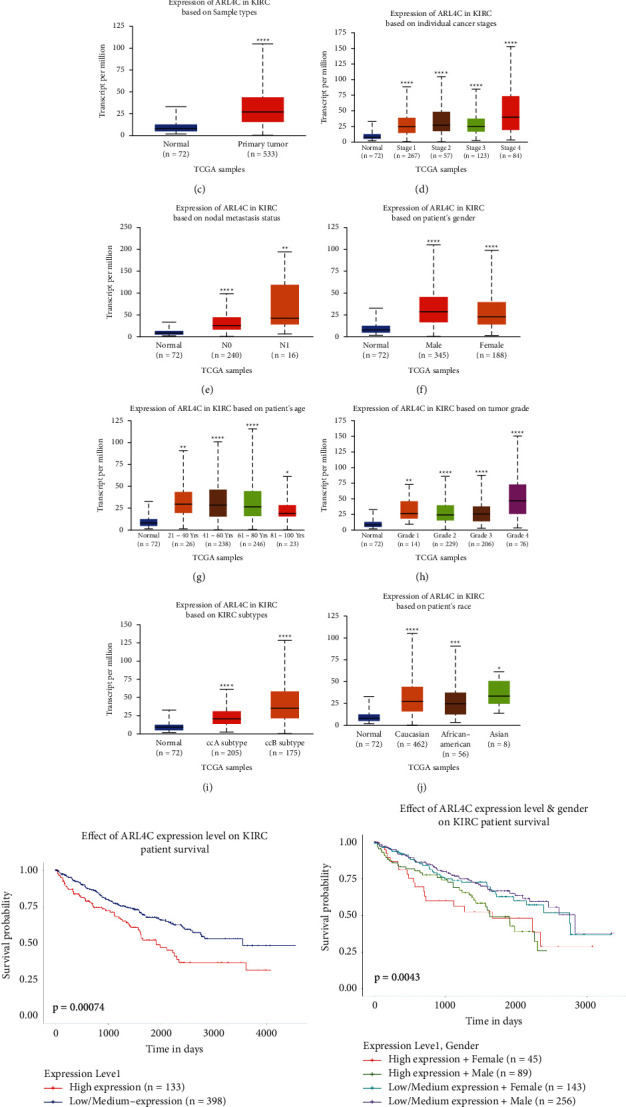
Expression of ARL4C in pan-cancer and renal cancer and its relationship with the clinical characteristics of KIRC patients based on TCGA database. (a) ARL4C expression in pan-cancer was explored using TCGA database. (b) Comparison of ARL4C expression in pan-cancer tumor tissues and adjacent normal tissues. Red represents tumor tissues, and blue represents normal tissues. (c) The UALCAN website was applied to compare the expression of ARL4C mRNA in primary KIRC tissues versus normal kidney tissues. (d–j) The relationship between ARL4C expression and different cancer stages, lymph node metastasis status, patient's sex, patient's age, tumor grade, KIRC subtype, and patient's race is based on the UALCAN website. (k) Effect of ARL4C mRNA expression on KIRC patient survival. (l) Effect of ARL4C expression and sex on KIRC patient survival. (m) Effect of ARL4C expression and tumor grade on KIRC patient survival. (n) Effect of ARL4C expression and race on KIRC patient survival. ^*∗*^*P* < 0.05, ^*∗∗*^*P* < 0.01, ^*∗∗∗*^*P* < 0.001, and ^*∗∗∗∗*^*P* < 0.0001.

The UALCAN website is a powerful tool for online bioinformatics analysis using multiple public databases, including TCGA. This online analysis tool was used to explore the expression of ARL4C in different cancers and its relationship with the clinical characteristics of KIRC patients. The results showed that ARL4C was differentially expressed in various cancers (Figure 3(a)). The differential expression analysis between cancer tissues and their corresponding adjacent normal tissues showed that ARL4C expression in KIRC tissues was higher than that in adjacent kidney tissues. Similarly, the expression of ARL4C in cervical squamous cell carcinoma and endocervical adenocarcinoma (CESC), cholangiocarcinoma (CHOL), esophageal carcinoma (ESCA), kidney renal papillary cell carcinoma (KIRP), pheochromocytoma and paraganglioma (PCPG), sarcoma (SARC), and other cancer tissues increased, whereas the expression of ARL4C in BRCA (breast invasive carcinoma) and kidney chromophobe (KICH) was decreased (Figure 3(b)). We also explored the effect of ARL4C expression on various clinical features of KIRC, and we found that ARL4C was not only differentially expressed in normal tissues versus primary tumors but also correlated with the cancer stage of patients, lymph node metastasis status, sex, age, tumor grade, KIRC subtype, and race (Figures 3(c)–3(j)). More importantly, high expression of ARL4C suggested a poor survival prognosis in KIRC patients (*P*=0.00074) (Figure 3(k)). Moreover, the expression of ARL4C and the sex of the patient together affected the prognosis of KIRC patients (*P*=0.0043) (Figure 3(l)). We also found that the level of ARL4C and patient tumor grade together correlated with the prognosis of patients (*P* < 0.0001) (Figure 3(m)). Finally, the level of ARL4C and patient's race were associated with the prognosis of KIRC patients (*P* < 0.0025) (Figure 3(n)).

### 3.4. qRT-PCR and Immunohistochemistry Confirm the Differential Expression of ARL4C in KIRC Tissues

**Figure 4:**
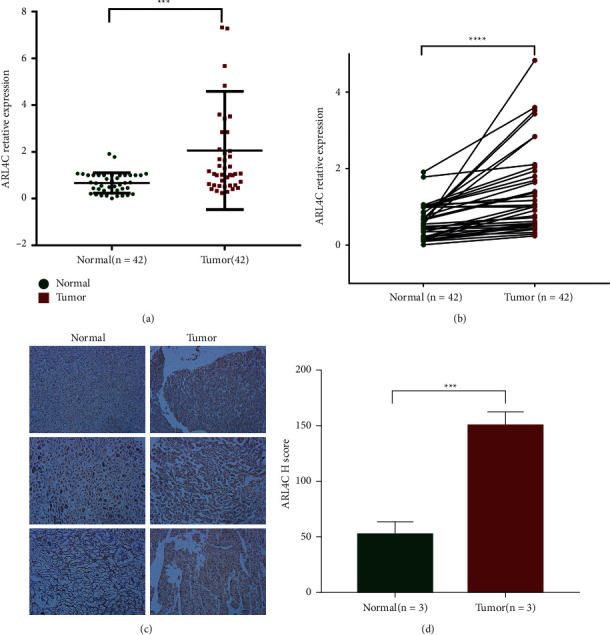
qRT-PCR and immunohistochemistry of ARL4C expression in KIRC. (a-b) ARL4C mRNA expression in KIRC and normal kidney tissues (*n* = 42) was determined using qRT-PCR. (c-d) Immunohistochemical analysis was performed on KIRC and normal kidney tissues (*n* = 3) to determine the expression of ARL4C, and histograms of H-scores for three representative samples were generated. ^*∗∗∗*^*P* < 0.001, ^*∗∗∗∗*^*P* < 0.0001.

In addition to exploring the expression and role of ARL4C in KIRC tissues using bioinformatics analysis, we further collected 42 pairs of ccRCC and adjacent normal tissues. We determined the mRNA expression of ARL4C in clinical tissues using qRT-PCR (Figure 4(a)). The results showed that the expression of ARL4C was significantly increased in the ccRCC tissues compared with that in the adjacent normal tissues, which confirms the accuracy of our bioinformatics analysis results (Figure 4(b)). In addition, we randomly chose three pairs of ccRCC and adjacent normal tissues for immunohistochemical analysis, and histograms of the H-scores of these three typical samples were generated (Figure 4(c)). The results suggested that the protein expression of ARL4C in ccRCC tissues was consistent with the mRNA expression level (Figure 4(d)).

### 3.5. Expression of ARL4C in Normal Renal and ccRCC Cell Lines and Establishment of ARL4C Knockdown Renal Cancer Cell Lines

**Figure 5:**
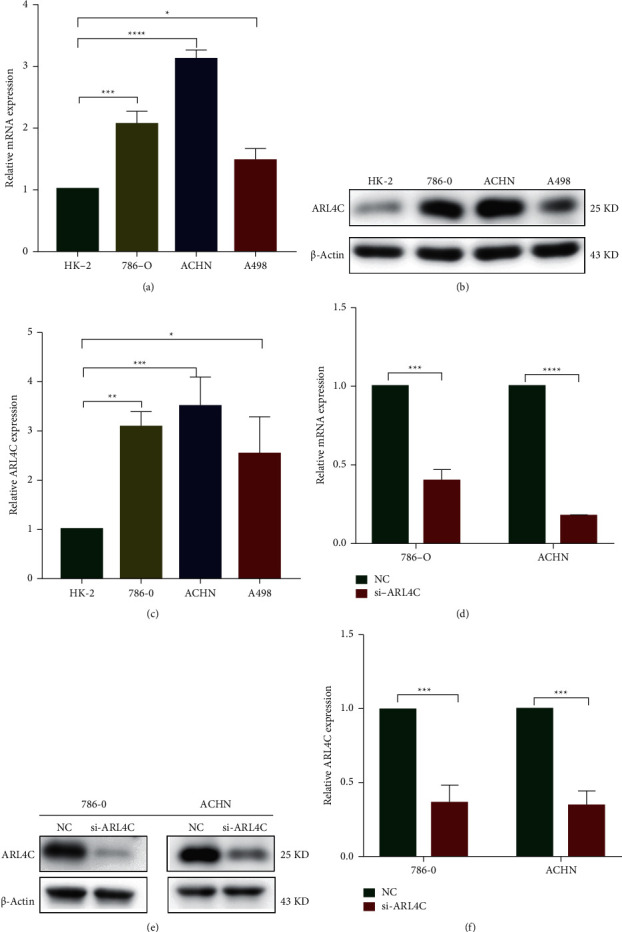
Expression of ARL4C in normal renal cells and ccRCC cell lines and establishment of ARL4C knockdown ccRCC cell lines. (a) ARL4C mRNA expression in normal kidney cells (HK-2) and different ccRCC cell lines (786-O, ACHN, and A498) was determined using qRT-PCR and quantified as a histogram. (b-c) Western blot showing the amount of ARL4C protein in HK-2, 786-O, ACHN, and A498 cells, represented quantitatively for the screened cell lines as a histogram. (d) qRT-PCR determination of the knockdown efficiency after transfection of negative control small interference (si-NC) and knockdown ARL4C small interference (si-ARL4C) into ACHN and 786-O cells. (e-f) Western blot of the expression of ARL4C protein in the si-NC and si-ARL4C groups in 786-O and ACHN cell lines, shown quantitatively as a histogram. ^*∗*^*P* < 0.05, ^*∗∗*^*P* < 0.01, ^*∗∗∗*^*P* < 0.001, and ^*∗∗∗∗*^*P* < 0.0001.

ARL4C mRNA and protein expression in normal kidney cells (HK-2) and three ccRCC cell lines (786-O, A498, and ACHN) were determined using qRT-PCR and western blot, respectively (Figures 5(a)–5(c)). The results showed that ARL4C mRNA and protein expression in the ccRCC cells were higher than those in HK-2 cells. The expression of ARL4C in 786-O and ACHN cell lines was higher than that in A498 cells. Therefore, 786-O and ACHN cell lines were selected for small interference transfection, and then two types of ARL4C knockdown renal cancer cell lines were established. Both qRT-PCR and western blotting were used to verify the efficacy of the knockdown (Figures 5(d)–5(f)).

### 3.6. Knockdown of ARL4C Inhibits Proliferation of 786-O and ACHN Cell Lines

**Figure 6:**
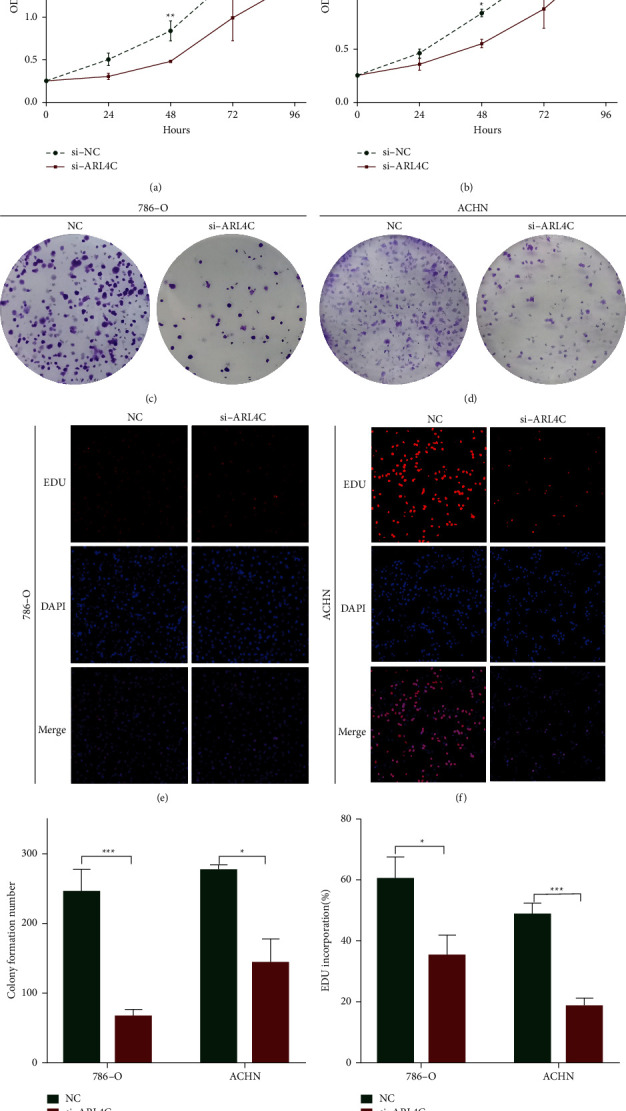
Knockdown of ARL4C inhibits proliferation of 786-O and ACHN cells. (a-b) or ACHN and 786-O cells, CCK-8 analysis was used to detect the OD values of the si-NC and si-ARL4C groups after 24, 48, 72, and 96 h, and a line graph was generated. (c-d) Colony formation assay was used to determine the proliferation of ACHN and 786-O cells in the si-NC and si-ARL4C groups. (e-f) EdU assay was used to determine the proliferation ACHN and 786-O cells in the si-NC and si-ARL4C groups. (g) The number of colonies was counted using Image-Pro Plus software and represented as a histogram. (h) EdU incorporation was calculated using Image-Pro Plus software and represented as a histogram. ^*∗*^*P* < 0.05, ^*∗∗*^*P* < 0.01, ^*∗∗∗*^*P* < 0.001, and ^*∗∗∗∗*^*P* < 0.0001.

To determine whether ARL4C knockdown affects the proliferative capacity of ccRCC cells, we first compared the cell proliferation of the si-NC group versus the si-ARL4C group using CCK-8 (Figures 6(a) and 6(b)) and colony formation assays (Figures 6(c) and 6(d)). The results indicated that the proliferative capacity of 786-O and ACHN cells was substantially reduced due to ARL4C knockdown (Figure 6(g)). EdU experiments showed that ARL4C knockdown significantly reduced the number of EdU-positive cells (Figures 6(e) and 6(f)), thus further demonstrating that ARL4C knockdown inhibited the proliferation of 786-O and ACHN cells (Figure 6(h)).

### 3.7. Knockdown of ARL4C Inhibits Migration and Invasion of 786-O and ACHN Cell Lines

**Figure 7:**
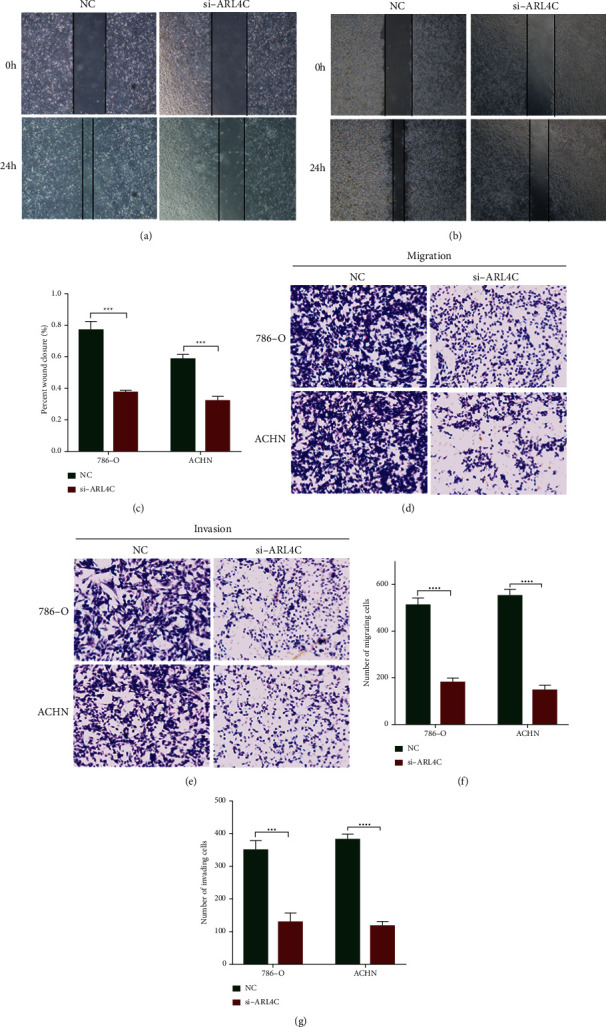
Knockdown of ARL4C inhibits migration and invasion of 786-O and ACHN cells. (a-b) Images were obtained using an optical microscope at 0 and 24 h after 786-O and ACHN cell monolayer “wounding.” (c) The degree of wound healing in the NC and si-ARL4C groups in ccRCC cells was quantified using Image-Pro Plus software. (d-e) The 786-O and ACHN cells that passed through or invaded the Transwell filter were stained and photographed using an inverted microscope. (f-g) The number of migrating and invading cells in the NC and si-ARL4C groups in ccRCC cells was calculated using Image-Pro Plus software and represented as histograms. ^*∗∗∗*^*P* < 0.001 and ^*∗∗∗∗*^*P* < 0.0001.

In addition to alteration of the proliferative capacity, altered cancer cell migration and invasion capabilities also affect the cancer prognosis. Wound healing and Transwell assays were used to investigate whether ARL4C affects the migration and invasion of 786-O and ACHN cells. We found that the wound healing ability of the si-ARL4C group of the two ccRCC cell lines was higher than that of the si-NC group, indicating that knockdown of ARL4C significantly inhibited the migration ability of renal cancer cells (Figures 7(a)–7(c)). Migration experiments further confirmed this finding (Figures 7(d) and 7(f)). In addition, the results of the invasion experiment indicate that the invasion by the two types of ccRCC cells was considerably inhibited following ARL4C knockdown, thus suggesting that the expression of ARL4C positively correlates with the invasive ability of ccRCC cells (Figures 7(e) and 7(g)).

### 3.8. ARL4C in Wnt/*β*-Catenin-Mediated Regulation of EMT in ccRCC

**Figure 8:**
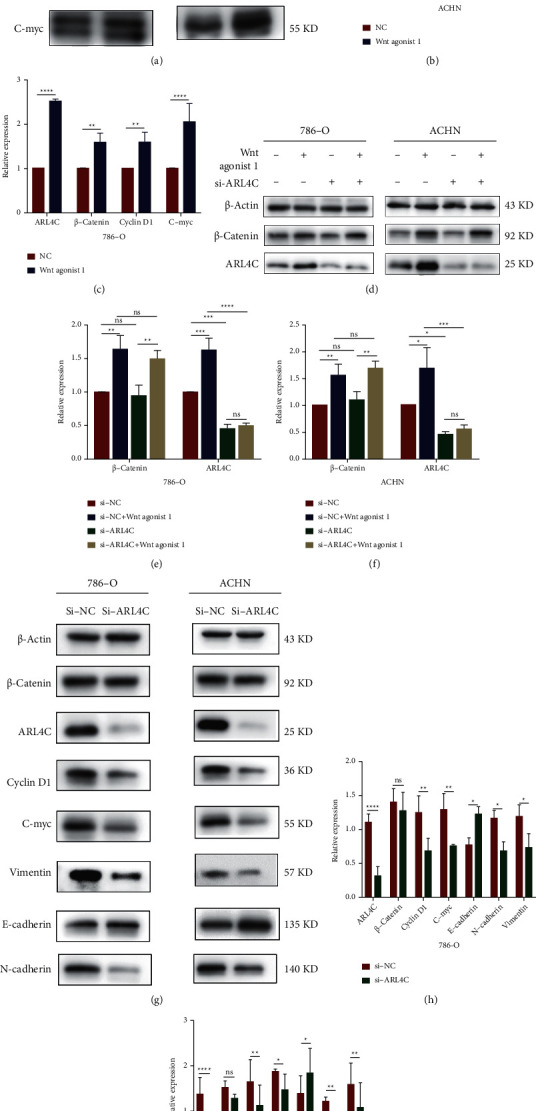
As a key effector of the Wnt/*β*-Catenin signaling pathway, ARL4C regulates EMT in ccRCC. (a–c) Western blot of the expression of ARL4C, *β*-catenin, cyclin D1, and c-myc proteins in 786-O and ACHN cells after adding Wnt agonist 1. (d-f) 786-O and ACHN cells were cultured with and without Wnt agonist 1 (Wnt agonist 1+/−), and ARL4C was then knocked down or not knocked down again (si-ARL4C/si-NC), followed by western blotting to determine the expression of ARL4C and *β*-catenin. (g-i) Expression of ARL4C, Wnt pathway-related proteins (*β*-catenin, cyclin D1, and c-myc), and EMT-related proteins (*E*-cadherin, *N*-cadherin, and vimentin) following knockdown of ARL4C in 786-O and ACHN cells. The above quantitative analysis of protein expression was normalized relative to *β*-actin levels. ^*∗*^*P* < 0.05^*∗∗*^*P* < 0.01, ^*∗∗∗*^*P* < 0.001, and ^*∗∗∗∗*^*P* < 0.0001.

**Figure 9:**
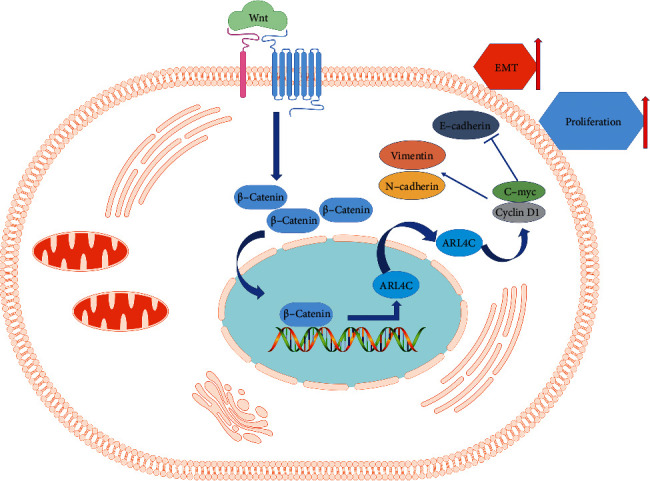
Schematic representation of ARL4C as a key molecule of the Wnt/*β*-catenin pathway regulating the EMT of ccRCC (further experimental confirmation is required).

Wnt agonist 1 activates the Wnt signaling pathway by affecting the transcriptional activity of *β*-catenin and TCF. First, we explored the changes in levels of specific proteins in 786-O and ACHN cells following exposure to Wnt agonist 1 using western blotting experiments (Figures 8(a)–8(c)). The results showed that the addition of Wnt agonist 1 increased the expression of *β*-catenin, as well as cyclin D1 and c-myc, which are Wnt signaling-dependent gene products. More importantly, the protein expression of ARL4C was significantly increased by exposure to Wnt agonist 1, thus suggesting that the expression of ARL4C in ccRCC cells is involved in the Wnt signaling pathway. To further verify that the Wnt/*β*-catenin signaling pathway regulates the protein expression of ARL4C in renal cancer cells, we cultured 786-O and ACHN cells with or without Wnt agonist 1 (Wnt agonist 1+/−) and ARL4C knockdown (si-ARL4C/si-NC). The results showed that after adding Wnt agonist 1, the expression of *β*-catenin increased, indicating that Wnt agonist 1 activates the Wnt signaling pathway in renal cancer cells by affecting *β*-catenin expression, namely, by Wnt/*β*-catenin signaling. In addition, after adding Wnt agonist 1, the expression of ARL4C protein was increased. However, the level of *β*-catenin was significantly altered with and without ARL4C knockdown, suggesting that ARL4C is a downstream factor in ccRCC that can be affected by altered Wnt/*β*-catenin signaling (Figures 8(d)–8(f)). After confirming that ARL4C protein is regulated by Wnt/*β*-catenin signaling, we knocked down ARL4C in ccRCC cells to further explore the changes in the expression of Wnt pathway-related proteins. We found that the expression of cyclin D1 and c-myc proteins, which are Wnt signaling-dependent genes, was significantly inhibited by ARL4C knockdown. Furthermore, our results showed that the knockdown of ARL4C resulted in the upregulation of E-cadherin in 786-O and ACHN cells, whereas the levels of *N*-cadherin and vimentin were reduced due to ARL4C knockdown. The above results indicate that the knockdown of ARL4C in these cells could inhibit EMT, thus revealing the molecular mechanism by which knockdown of ARL4C inhibited the proliferation, migration, and invasion abilities of 786-O and ACHN cells (Figures 8(g)–8(i)). The mechanism by which ARL4C affects ccRCC cells is summarized schematically in Figure 9.

## 4. Discussion

Global cancer research statistics show that, in 2020, the incidence and deaths of kidney cancer accounted for 2.2% and 1.8% of all cancer types, respectively [1]. RCC is the most common malignant tumor of the kidney and the most lethal urinary system tumor. There are several subtypes of RCC. The most commonly observed subtype in clinical practice is ccRCC, named for its cytoplasm rich in lipids and pathologically stained “clear” state [24]. The formation or prognosis of ccRCC caused by deletion or mutation of related molecules is widely recognized [25, 26]. These molecules play a biological role in promoting or inhibiting cancer by affecting the changes in the epigenetics, proteomics, and metabolomics of tumor cells [27]. Drug therapy specifically targeting the angiogenesis pathway has become the main treatment for advanced metastatic renal cancer [28]. However, tumor resistance caused by long-term administration of the targeting drugs is still one of the main reasons for the poor prognosis of clinically advanced metastatic renal cancer. Detailed exploration of the mechanisms underlying renal cancer, elucidation of the etiology of ccRCC, and discovery of new therapeutic targets have had significant clinical impacts in improving the survival rate of patients with advanced renal cancer.

The full-length coding sequence of ARL4C was first determined in human bladder epithelial cells by Jacobs et al. in 1999 [12]. ARL4C belongs to the superfamily of *G* proteins and plays a variety of biological functions in organisms. Previous studies have found that ARL4C can bind to *α*-tubulin and regulate the intracellular vesicular transport of transferrin, thereby affecting intracellular iron metabolism. Unlike the mechanism of action of other ARF family members, the activity of ARL4C does not depend on the binding of GTP or GDP [29]. Previous experiments found that ARL4C is not only regulated by liver *X* receptor (LXR)/retinoic acid *X* receptor agonists but also by cholesterol in macrophages, which means that ARL4C is a downstream effector of LXR in macrophages [30]. LXR is a key regulator of fatty acid synthesis and cholesterol transport, suggesting that ARL4C has potential functions in lipid metabolism. Our research on ccRCC has found that the LXR agonist LXR623 downregulates the low-density lipoprotein receptor (LDLR) protein and upregulates the expression of ABCA1, which leads to reduced levels of intracellular cholesterol and enhanced apoptosis in ccRCC. In contrast, the same effect does not appear to occur in normal renal cells [31]. The above studies suggest that ARL4C may promote the formation and progression of cancer by affecting the lipid metabolism of ccRCC cells. ARL4C acts as a common downstream factor of Wnt/*β*-catenin and EGF-Ras-MAPK signaling, and ARL4C activates Rac by upregulating ARF6 to inhibit the expression of Rho. The changes in the above pathways induce cytoskeletal rearrangements and morphological changes, which in turn affect the shape of epithelial tubular structures and promote cell migration. Changes in cell morphology can lead to the nuclear translocation of YAP and TAZ, thereby promoting cell proliferation. Further studies have found that endogenous Wnt leads to embryonic kidney branching morphogenesis in mice by affecting the expression of ARL4C, thereby revealing the role of ARL4C in kidney development [32]. The process of basic morphogenesis of most epithelial organs is called epithelial tubular morphogenesis (tubulogenesis). During tubulogenesis, epithelial cells migrate toward the surrounding mesenchymal tissue and proliferate, polarize, and differentiate to form tubule structures. Epithelial tissue architecture is largely stable after birth, but epithelial cells regain a high proliferative and invasive potential during tumor formation (tumorigenesis) [33]. Therefore, we believe that ARL4C affects tubulogenesis and may also be involved in epithelial tumorigenesis. Many previous studies confirmed that ARL4C is involved in the formation and progression of various cancers, and the functions and molecular mechanisms of ARL4C differ significantly according to the tumor type. In addition, a study found that the distribution of ARL4C in human tissues has apparent tissue differences. For example, ARL4C mRNA expression in the brain is high, but it is lower in many organs such as the spleen, stomach, intestine, and uterus than that in other organs [12]. We believe that the differences in tissue distribution of ARL4C may lead to different functions and molecular mechanisms of ARL4C in different tissues. Fujii et al. found that ARL4C acts as a common downstream effector of Wnt/*β*-catenin and EGF-Ras-MAPK signaling in colorectal and lung cancers. Correlation of ARL4C expression with clinical features of colorectal or lung cancer indicates that ARL4C expression does not change with tumor *T* grade or lymph node metastasis grade, indicating that it is involved in the occurrence rather than the development of such cancers. Rac is activated as a downstream effector of ARL4C, which reduces the expression of Rho and, in turn, affects the invasion of cancer cells. The proliferation of cancer cells is related to the nuclear localization of YAP/TAZ caused by ARL4C [34]. In addition to the mutational deletion of genes involved in tumorigenesis, epigenetic modifications can also be involved in the expression of related molecules that affect the development of tumors [35]. Another study found that the AKT pathway is an important regulatory pathway for ARL4C expression in lung cancer cells, and the authors showed that the chemotherapeutic drug hydroxycamptothecin (HCPT0) could be used to treat the lung adenocarcinoma by targeting the expression of ARL4C [36].

The Wnt signaling pathway is a key mechanism in human embryogenesis and adult homeostasis. The canonical Wnt signal is transmitted to *β*-catenin through Frizzled (FZD) family receptors and LRP5/LRP6 coreceptors. *β*-Catenin is then activated by related enzymes and localized and transported into the nucleus for accumulation [37]. Nuclear *β*-catenin and other key molecules constitute the TCF/LEF-*β*-catenin-Legless-PYGO nuclear complex, which activates the transcription of downstream target genes [38]. Several studies suggest that continued activation of the Wnt/*β*-catenin signaling pathway is a prerequisite for the constant renewal and proliferation of tumor cells [39–41]. Targeting a critical molecule of the Wnt/*β*-catenin pathway and selectively blocking signal transduction can achieve the purpose of treating tumors [42]. With the development and application of molecularly targeted drugs, many studies are focused on developing targeted clinical medicines and experimental reagents to regulate different components of the Wnt/*β*-Catenin signaling pathway [43]. Wnt agonist 1 is an agent that activates the Wnt signaling pathway as it can penetrate the cell membrane. By activating the Wnt signaling pathway, it increases the transcriptional activity of *β*-catenin and TCF [44]. In addition, related studies have found that Wnt agonist 1 can enhance *β*-catenin translocation to the nucleus in gastric cancer, thereby increasing the expression of *β*-catenin and Wnt signaling-dependent genes [45]. In recent years, there has been increasingly extensive exploration of the function of Wnt/*β*-catenin in the initiation and progression of different cancers [46], and cyclin D1 and c-myc, which are downstream targets of Wnt/*β*-catenin signaling, have been shown to affect tumor proliferation and metastasis [46, 47]. Serpin H1 acts on EMT as a critical factor in the Wnt/*β*-catenin pathway, affecting cell survival, invasion, and migration in gastric cancer [48]. In colorectal cancer, Sec62 is activated by METTL3-mediated m6A modification, which enhances Wnt signaling by binding to *β*-catenin, thereby affecting the prognosis of colorectal cancer [49]. A recent study found that E7386 selectively inhibits Wnt/*β*-catenin signaling by inhibiting the formation of complexes involving *β*-catenin and CBP [50]. It has been widely used to treat different cancers due to its specific effects. ALK combined with N-myc (ALK F1174 C/N-myc) co-activates Wnt/*β*-catenin signaling, and Wnt inhibitors targeting these entities can inhibit the growth and metastasis of neuroendocrine prostate cancer and neuroblastoma [51]. LGK974 is a specific PORCN inhibitor, and it significantly inhibits the proliferation and EMT of ccRCC cells. This effect was mediated by LGK974 affecting the Wnt/*β*-catenin pathway, thus suggesting that targeting the Wnt/*β*-catenin pathway is likely to have a significant auxiliary impact on the treatment of renal cancer [52]. Previous research found that the Wnt/*β*-catenin signaling pathway can alter tubulogenesis by affecting the expression of ARL4C [32]. During tubulogenesis, epithelial cells migrate, proliferate, polarize, and differentiate toward the surrounding mesenchymal tissue, thereby forming tubule structures. However, tumorigenesis restores proliferative and invasive potential by transforming epithelial cells into mesenchymal tissue. Therefore, we believe that ARL4C acts as an essential regulator that affects the EMT in ccRCC, which is involved in the development of renal tumors.

The TCGA database contains data on 539 KIRC tumor tissues and 72 normal kidney tissues. We downloaded the mRNA expression data and clinical characteristics of these samples and identified the differential expression of 22 ARL genes in KIRC versus normal kidney tissues. We then applied univariate Cox retrospective analysis and Lasso regression analysis to evaluate the role of each ARL gene on the prognosis of renal cancer. Based on the above bioinformatics analysis, we obtained a preliminary indication that ARL4C may be an independent risk factor affecting KIRC. The subsequent survival curve revealed that the prognosis of KIRC patients in the high-score group was poor. We downloaded related drugs from the GDSC database and evaluated the correlation between mRNA expression of ARL genes and GDSC drug sensitivity. The results showed that ARL4C may be a potential target for KIRC-targeted drug therapy. Methylation is an important epigenetic modification in oncology, and the biological function of specific tumor-related molecules changes after modification by methylation, which in turn affects the fate of tumors. This study showed that methylation of ARL4C was down-regulated in KIRC tumors, thus suggesting that KIRC may involve demethylation. We then explored the correlation between various immune cells and mRNA expression of ARLs in KIRC patients. We found that ARL4C expression was related to the infiltration of various immune cells, thus suggesting that ARL4C may play a role in selecting immune cells that infiltrate the tumor microenvironment in KIRC tumors through a specific mechanism. Finally, the results of our analysis showed infiltration of various types of immune cells in different tumor types, and we found that infiltration of various immune cells may affect the occurrence and progression of KIRC tumors. We used the UALCAN website to analyze the expression of ARL4C mRNA in human ccRCC tissues and its association with clinical features. Compared with normal tissues, the expression of ARL4C in human ccRCC tissues was significantly higher, and its expression was significantly related to various clinical features. In particular, the high expression of ARL4C in KIRC suggested a poor prognosis.

In this study, we further collected 42 pairs of ccRCC and adjacent normal tissues, determined the expression of ARL4C mRNA in these clinical tissues using qRT-PCR, and collected three pairs of ccRCC and adjacent normal tissues for immunohistochemical analysis. The results showed that the expression of ARL4C in the ccRCC tissues was significantly higher than that in adjacent normal tissues. To further study the biological role of ARL4C in ccRCC, we used 786-O and ACHN cell lines for small interference transfection after screening and established two ARL4C knockdown renal cancer cell lines. Our study showed that downregulation of ARL4C significantly inhibited the proliferation, migration, and invasion abilities of ccRCC cells. Further research found that the activation of the Wnt/*β*-catenin pathway promoted the expression of ARL4C. As a key component of the Wnt signaling pathway, ARL4C upregulates the expression of cyclin D1 and c-myc, thereby affecting the EMT of ccRCC cells and promoting ccRCC. The above studies shed light on the mechanisms underlying the occurrence and development of ccRCC. ARL4C acts as an oncogene in ccRCC, and its in-depth study can lead to the identification of new targets and prognostic markers for gene therapy of renal cancer.

## 5. Conclusions

Our study of ARL4C found that the ARL4C levels were elevated in ccRCC tissues and that high expression predicted poor prognosis in ccRCC. Knockdown of ARL4C inhibited the proliferation, colony formation, migration, and invasion of renal cancer cells. The above changes in cancer characteristics were due to the changes in EMT of renal cancer cells caused by the knockdown of ARL4C. Mechanistically, we believe that the activation of the Wnt/*β*-catenin pathway promotes the expression of ARL4C. This then upregulates the expression of Wnt pathway-related genes coding for cyclin D1 and c-myc, which in turn affects the EMT of ccRCC cells. The main limitation of this study was that it did not involve a more in-depth study of the specific regulatory mechanism of ARL4C in the Wnt/*β*-catenin pathway. Our research shows that ARL4C expression is increased in ccRCC compared with that in normal kidney tissue, which promotes the development of ccRCC. Research on targeted drugs that inhibit ARL4C may provide new options for clinical KIRC treatment.

## Data Availability

Bioinformatic analyses and experimental data supporting our research findings can be obtained from the corresponding authors with reasonable justification.

## Abbreviations

KIRC: Kidney renal clear cell carcinoma
ccRCC: Clear cell renal cell carcinoma
Rho: Rhodopsin
Ras: Resistance to audiogenic seizures
Rab: Ras-related GTP-binding protein
Arf: ADP-ribosylation factor
TCGA: The cancer genome atlas
EMT: Epithelial-mesenchymal transition
LASSO: Least absolute shrinkage and selection operator
GBM: Glioblastoma multiforme
GDSC: Genomics of drug sensitivity in cancer
CESC: Cervical squamous cell carcinoma and endocervical adenocarcinoma
CHOL: Cholangiocarcinoma
ESCA: Esophageal carcinoma
KIRP: Kidney renal papillary cell carcinoma
PCPG: Pheochromocytoma and paraganglioma
SARC: Sarcoma
BRCA: Breast invasive carcinoma
MAPK: Mitogen-activated protein kinase.
